# Supplementary material for: Sustainability of newborn screening for sickle cell disease in resource-poor countries: A systematic review
Source: PLoS One. 2024 Sep 6;19(9):e0305110. doi: 10.1371/journal.pone.0305110 (PMC11379310; doi:10.1371/journal.pone.0305110)
Supplement: S1 Table — (DOCX) [file pone.0305110.s002.docx]

| Questions | Archer et al., 2022 | Lewis HSU et al., 2018 | El-Haj N, Hoppe CC2018 | Rinke et al., 2016 | Coppinger et al., 2019 | Catherine et al., 2019 | Nnodu et al .,2020 | Green et al., 2016 | Bukini et al .,2021 | Segbefia et al., 2021., | Questions | Okeke et al .,2022 |
| --- | --- | --- | --- | --- | --- | --- | --- | --- | --- | --- | --- | --- |
| Was there a clear statement of the aims of the research? | Y | y | y | Y | Y | Y | Y | Y | Y | Y | . Was there a clear question for the study to address - | Y |
| Is a qualitative methodology appropriate | Y | y | y | Y | Y | Y | Y | Y | Y | y | Was there a comparison with an appropriate reference standard? | Y |
| Was the research design appropriate to address the aims of the research? | Y | y | y | Y | ? | Y | Y | Y | y | Y | Did all patients get the diagnostic test and reference standard. | Y |
| Was the recruitment strategy appropriate to the aims of the research? | Y | y | y | Y | Y | Y | Y | y | Y | Y | Could the result of the test have been influenced by the results of the reference standard | N |
| Was the data collected in a way that addressed the research issue | Y | y | ? | y | Y | y | y | y | y | y | Is the disease status of the tested population clearly described | y |
| Has the relationship between researcher and participants been adequately considered | ? | ? | ? | ? | ? | ? | ? | ? | ? | ? | Were the methods for performing the tets described in sufficient detail  Are the sensitivity and specificity and/or likelihood ratios presented | Y  Y |
| Have ethical issues been taken into consideration? | N | ? | N | N | N | Y | N | N | N | ? | Are there confidence limits | Y |
| Was the data analysis sufficiently rigorous? | Y | y | N | Y | Y | Y | Y | Y | Y | Y | Can the results be applied to your patients/ the population of interest? | ? |
| Is there a clear statement of findings? | Y | y | y | Y | y | y | Y | y | y | Y | Can the test be applied to your patient or population of interest | Y |
| Did the researcher discuss the contribution the study makes to existing knowledge? | y | y | y | y | y | y | y | y | y | y | Were all outcomes important to the individual or population considered | Y |
| Score out of 10 | 8 | 8 | 6 | 8 | 7 | 8 | 8 | 8 | 8 | 8 | Score out of 11 | 10 |

Y= Yes N=No ?= Can’t tell’
