# Supplementary material for: Sustainability of newborn screening for sickle cell disease in resource-poor countries: A systematic review
Source: PLoS One. 2024 Sep 6;19(9):e0305110. doi: 10.1371/journal.pone.0305110 (PMC11379310; doi:10.1371/journal.pone.0305110)
Supplement: Attachment — Submitted filename: Manuscript Review and Evaluation Pone.docx [file pone.0305110.s003.docx]

**Manuscript Review and Evaluation**

The manuscript provides a comprehensive overview of the challenges and potential strategies for sustainable implementation of Newborn Screening (NBS) programs for Sickle Cell Disease (SCD) in low-income, high-burden regions, particularly in African countries. The study amalgamates diverse sources, offering a detailed insight into the multifaceted issues hindering the efficacy of NBS programs and proposes various approaches to overcome these hurdles.

**Strengths:**

Comprehensive Analysis: The manuscript covers a broad spectrum of challenges, from economic limitations to inadequate resources and geographic constraints, providing a holistic view of the obstacles.

Solutions-Oriented Approach: It suggests pragmatic and contextually relevant solutions, emphasizing early government engagement, community advocacy, technological utilization, and policy alignment.

Integration of Multiple Sources: The inclusion of diverse references and studies enriches the manuscript, supporting the discourse with extensive evidence.

**Areas for Consideration:**

Limited Comparative Analysis: The scarcity of studies on NBS sustainability in Africa hinders the ability to present comparative sustainability approaches across different regions. Further analysis in this aspect could enhance the manuscript's depth.

In-Depth Case Studies: Incorporating detailed case studies or real-life examples of successful NBS implementation could strengthen the practicality of the proposed strategies.

**Concluding Insights:** The conclusion, though comprehensive, would benefit from a more specific delineation of actionable steps for immediate implementation.

**Overall Assessment:**

The manuscript offers a comprehensive understanding of the challenges and potential strategies for sustainable NBS implementation in low-income, high-burden regions. Despite limitations in comparative analysis and the need for more concrete conclusions, its relevance and the significance of proposed solutions make it a valuable resource in the realm of public health research.

**Recommendation:**

Consider further comparative analysis, inclusion of practical case studies, and a more specific and actionable conclusion to enhance the manuscript's impact and readability. With these enhancements, the manuscript will be a strong contribution to the field of public health research.
