# Supplementary material for: Sustainability of newborn screening for sickle cell disease in resource-poor countries: A systematic review
Source: PLoS One. 2024 Sep 6;19(9):e0305110. doi: 10.1371/journal.pone.0305110 (PMC11379310; doi:10.1371/journal.pone.0305110)
Supplement: Attachment — Submitted filename: Response to Reviewers..docx [file pone.0305110.s004.docx]

Centre of Excellence for Sickle Cell Disease Research and Training, University of Abuja (CESRTA), Abuja, Nigeria.

14^th^ March 2024

The Editor in Chief,

Plos One Publishers,

Sir,

**RESPONSE TO REVIEWERS**

Please find below our response to the comments made by the reviewers.

**Is the manuscript technically sound, and do the data support the conclusions?**

We made manual searches and included two more articles. We also created a chart containing article serial number corresponding with the table on the extraction form.

**Has the statistical analysis been performed appropriately and rigorously?**

Statistical analysis has been performed more rigourously; We carried out a fresh extraction of data from all the articles used for the review. We used inductive and deductive codes to arrive at 9 themes this time instead of eight as earlier stated. We created a table of frequency for the various articles within various themes, as can be seen below.

Extracted aspects of sustainability	Article Serial number corresponding with Table1Complete integration of services into national health care system	1,2,3,4,5,6, 7,8,9,10,11,

Suitable National SCD protocols	2, 5, 6,9,10,8,2,7,1,11

Expanding NBS for SCD to include other diseases	2,5,6,7,8,9

Efficient public health interventions	9, 4, 5, 6, 11,7,10

Quality improvement collaboration	3,2, 9,10

Data capture and management	1,2,3,6,7,9,4,10

Targeted NBS	1,2,6,7,9,10

Advocacy	1, 5, 6,7,8,10,11

Funding	1,7,9,4

The numbering. Are the serial numbers of the various articles in the extraction form.

**Have the authors made all data underlying the findings in their manuscript fully available?**

All the data has been made available.

**Is the manuscript presented in an intelligible fashion and written in standard English?**

Major edits and paraphrasings have been done on the article.

**Incorporating detailed case studies or real-life examples of successful NBS implementation could strengthen the practicality of the proposed strategies**.

Two more articles were added, one was the history of NBS for SCD in the USA

**The conclusion, though comprehensive, would benefit from a more specific delineation of actionable steps for immediate implementation**.

Added The implementation of the use of dried blood spots on HemoTypeSC could be a game changer in terms of scaling up and expanding the newborn screening program in Sub-Saharan Africa, among other action points. The model adopted by America was added.

**However, it was strange to find that the authors could ONLY find 9 studies to be eligible.**

Newborn screening is still sruggling in Africa, as such there isn’t much success stories we wanted to focus on Africa because of her unique difficulties and challenges. The limitations of this study were paucity of published articles on sustainability. We added two more articles after manual searches. We now have a total of eleven articles instead of nine. [https://www.tandfonline.com/doi/full/10.1080/13645579.2015.1005453](https://www.tandfonline.com/doi/full/10.1080/13645579.2015.1005453) recommended 10 articles as minimum for qualitative studies.

**No References are provided, when CASP was introduced in Page 6.**

The CASP reference has been added

Best regards,

Dr Chinwe Okeke
